# Supplementary material for: ComFC mediates transport and handling of single-stranded DNA during natural transformation
Source: Nat Commun. 2022 Apr 12;13:1961. doi: 10.1038/s41467-022-29494-z (PMC9005727; doi:10.1038/s41467-022-29494-z)
Supplement: Supplementary file 1 — Supplementary information [file 41467_2022_29494_MOESM1_ESM.pdf]

Supplementary Information

ComFC mediates transport and handling of single-stranded DNA during natural transformation

Prashant P. Damke, Louisa Celma, Sumedha Kondekar, Anne Marie Di Guilmi, Stéphanie Marsin, Jordane Dépagne, Xavier Veaute, Pierre Legrand, Hélène Walbott, Julien Vercruyssen, Raphaël Guérois, Sophie Quevillon-Cheruel and J. Pablo Radicella

Other Supplementary Information for this manuscript includes the following:

Movies S1 to S3

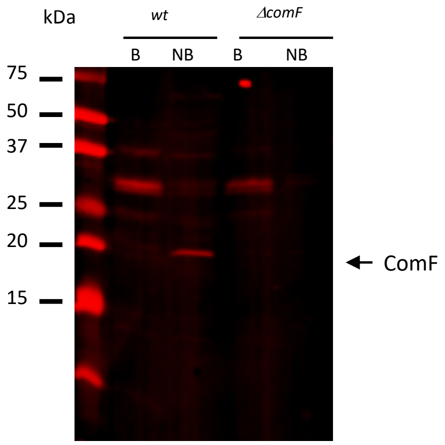

Supplementary Figure 1.
ComF(C) detection by immunoblotting. Total extracts from wild-type (*wt*) and *comFC* mutant *(comF*) *H. pylori* strains. Samples were either boiled (B) or not boiled (NB) prior gel loading. ComF is specifically detected in the NB condition. Blot is representative of two experiments.

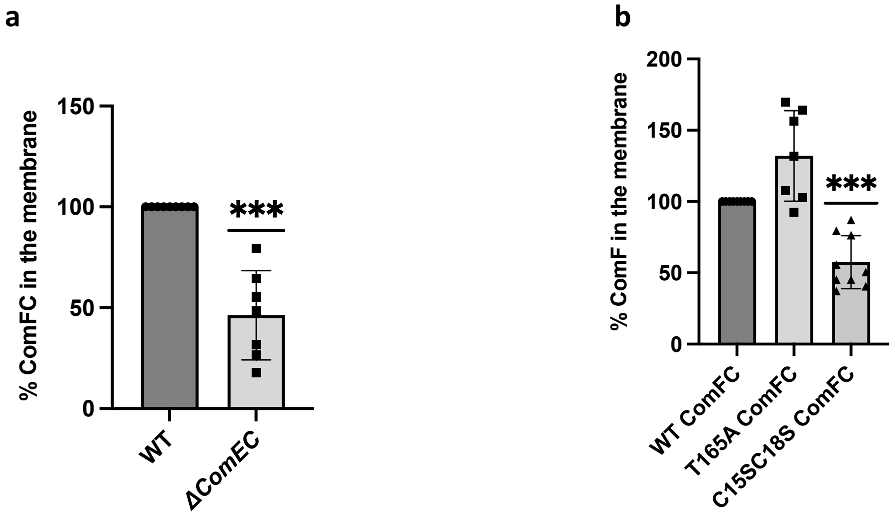

Supplementary Figure 2.
ComFC association with the membrane. (a) Quantification of Western blots comparing the amount of ComFC in membrane fraction of wild-type and *ΔcomEC* strains. Data are from at least three independent fractionations. n = 7 for each genotype (b) Quantification of Western blots comparing the wild-type and mutant ComFC present in the membrane fraction (n = 9 for WT ComFC, n = 7 for T165A ComFC and n = 9 for C15S C18S ComFC). Quantification was carried out by normalising the ComFC band intensity to that of the MotB band to take account of the membrane fraction recovery efficiency. Values for the wild-type strain (panel 2a) and wild-type protein (panel 2b) were set to 100%. Bars correspond to the mean +/- SD. *** p < 0.001 with respect to *wt* defined as 100% (One sample t test)

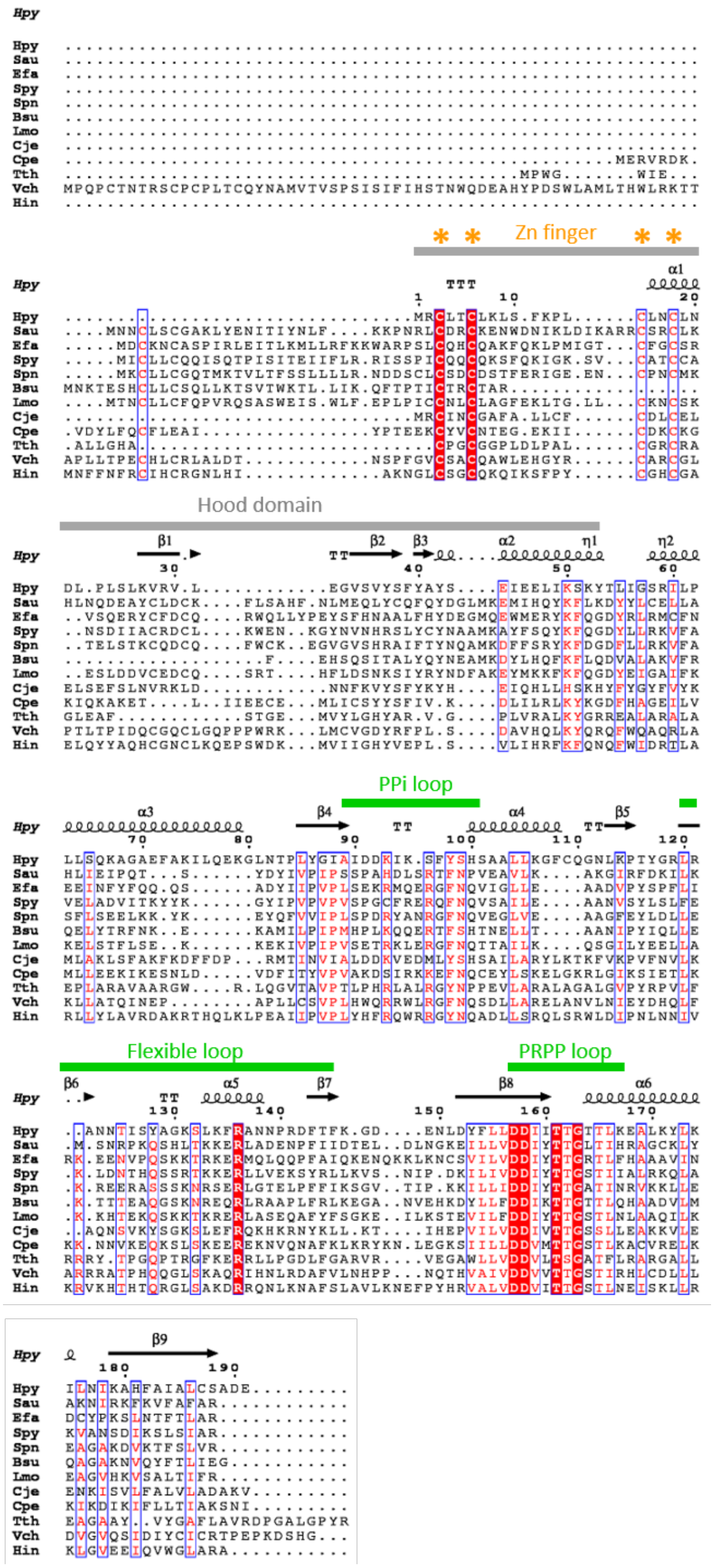
Zn finger
Hood domain
PPi loop
Flexible loop
PRPP loop

Supplementary Figure 3.

Sequence alignment of the ComFC proteins from gram(-) and gram(+) bacteria
The multialignment was generated by Clustalw2 (32). The figure was generated using ESPRIPT (33). The secondary structure elements of ComFC are in black on the top of the multialignment. Grey and green bars respectively localize the Hood domain and the three PRTase-loops. The 4 cysteine residues of the Zn-finger are indicated by orange stars. Hpy: *Helicobacter pylori*, Sau: *Streptococcus aureus*, Efa: *Enterococcus faecalis*, Spy: *Streptococcus pyrogenis*, Spn: *Streptococcus pneumonia*, Bsu: *Bacillus subtilis*, Lmo: *Listeria monocytogenes*, Cje: *Campylobacter jejuni*, Cpe: *Clostridium perfringens*, Tth: *Thermus thermophilus*, Vch: *Vibrio cholerae*, Hin: *Haemophilus influenzae*.

Supplementary Figure 4.

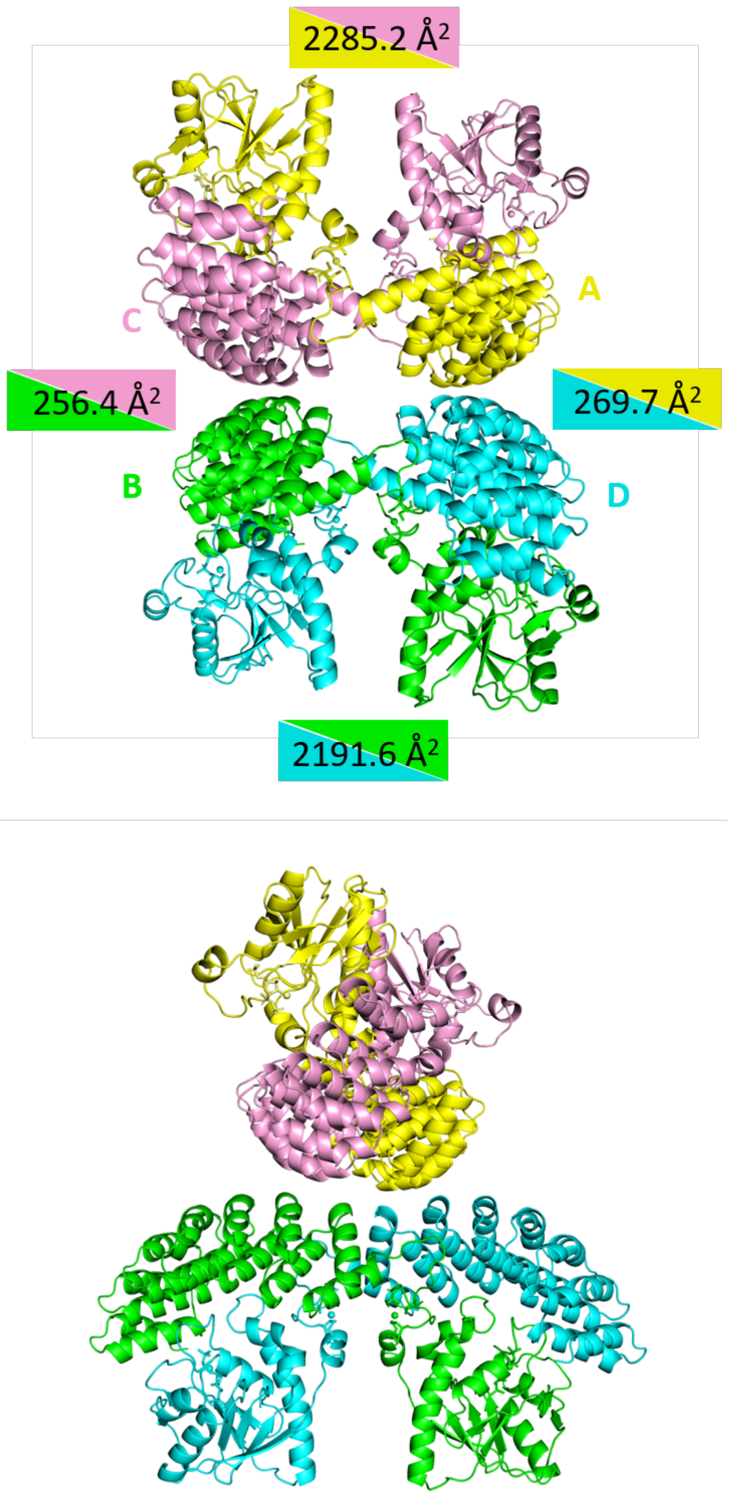

a. Two views of the crystal structure of the 4 molecules of the αRep-HpComFC fusion, forming 2 domain-swapped dimers in the asymmetric unit. The two protein fusions of the first dimer are in green and blue (colored as in Fig. 5), the two others are in pink and yellow. The PDBePISA server calculates the interface areas.

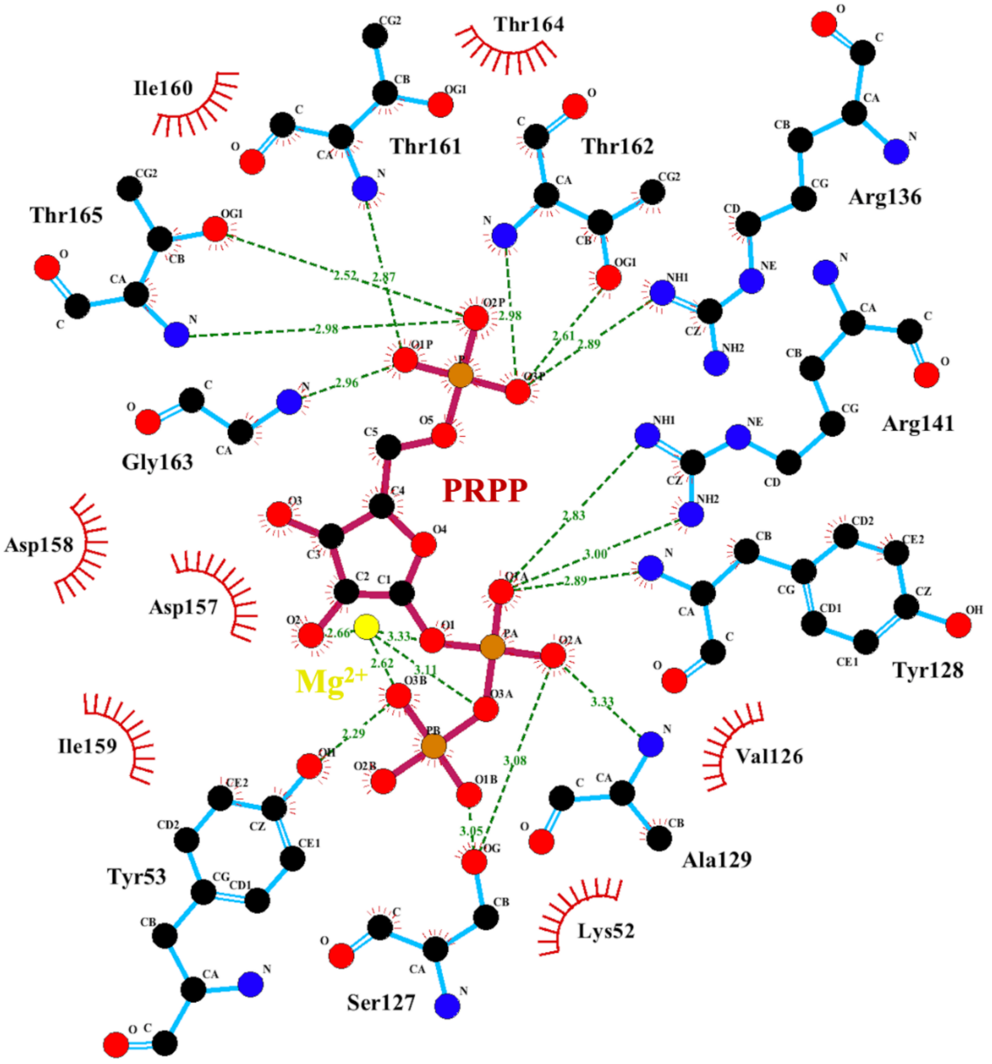

b. **ComFC binds the PRPP** (in red sticks) and Mg^{2+} ion (in yellow circle) through hydrogen bonds (green dotted lines, involved residues in cyan sticks) and hydrophobic forces (red half circles and dashes). The PRPP and the flexible loops are involved. The 9 residues of the PRPP loop are the characteristic motif of the PRTase family. 5 residues of the flexible loop can close the active site pocket to sequester the PRPP. Two other residues (K52 and Y53) located between helixes α2 and α3 are also involved in the binding of the PRPP.

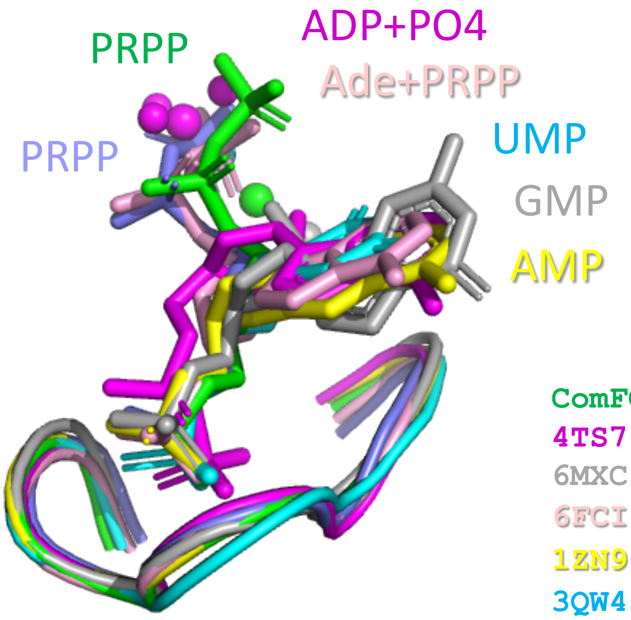

PRPP loops

```
ComFC     157-DDIITTGTTL-167
4TS7       95-DDITDTGDSI-105
6MXC      185-EDIADTGRTM-195
6FCI      126-DDLLATGGTM-136
1ZN9      127-DDLLATGGTM-137
3QW4      372-DDLVSTGETK-382
4RV4      144-EDLISTGGSA-154
              ::   :* :
```

c. Structural alignment of the PRPP loops of various PRTases co-crystallysed with ligands and Mg ions.

PDB ID 4TS7 : Adenine Phosphoribosyltransferase from *Sulfolobus solfataricus*
PDB ID 6MXC : Hypoxanthine-guanine phosphoribosyltransferase from *Trypanosoma brucei brucei*
PDB ID 6FCI : Adenine Phosphoribosyltransferase from Human
PDB ID 1ZN9 : Adenine Phosphoribosyltransferase from Human
PDB ID 3QW4 : UMP synthase from *Leishmania donovani*
PDB ID 4RV4 : Orotate phosphoribosyltransferase from *Bacillus anthracis*

PRPP : phosphoribosylpyrophosphate
UMP : uridine-5'-monophosphate
GMP : guanosine-5'-monophosphate
AMP : adenine-5'-monophosphate
Ade : Adenine

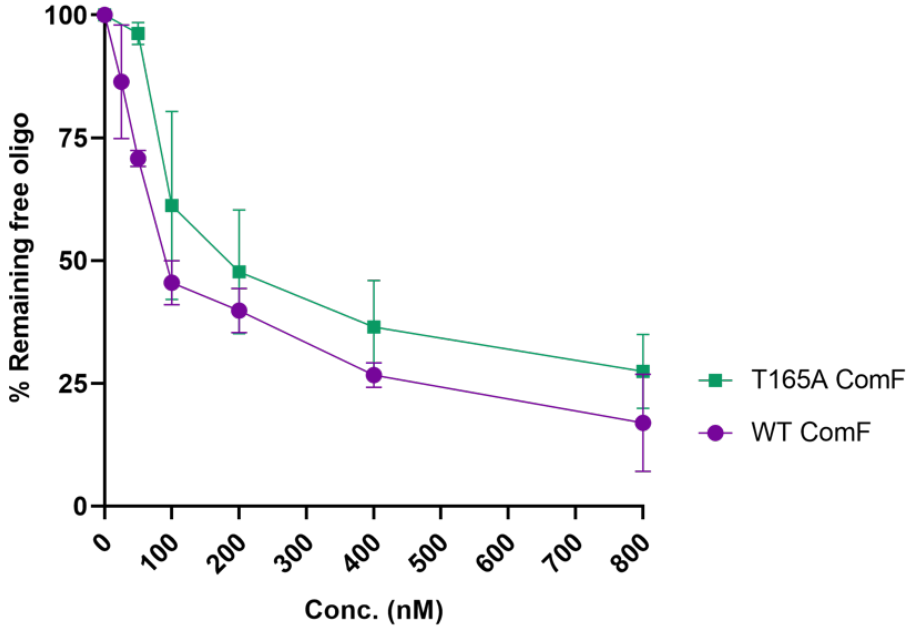

Supplementary Figure 5.

Comparison of DNA binding affinities of WT ComFC with ComFC T165A. No significant difference was observed between the binding of WT and T165A ComF proteins to ssDNA. Quantification of three independent electrophoretic mobility shift assays was performed using Image Studio software. Data corresponds to the Mean +/- SD.

Table S1.

Table S1.

Approximate melting temperature (^{o}C) of wild-type ComFC and ComFC-T165A with or without added ligand

<

Ligand	Wild-type ComFC	ComFC-T165A
AMP (0)	45.7	42.1
AMP (5mM)	55.1	43.7
AMP (10 mM)	55.0	45.2
ADP (0)	45.0	41.2
ADP (5mM)	51.4	44.6
ADP (10 mM)	51.6	42.9
ATP (0)	46.7	41.4
ATP (5mM)	45.0	41.2
ATP (10 mM)	45.8	41.5
Ribose-5-Phosphate (0)	44.9	41.3
Ribose-5-Phosphate (5 mM)	48.2	42.0
Ribose-5-Phosphate (10 mM)	48.2	41.8

Table S2.

Strains used in this study

**Strain**	**Genotype**	**Source**
LR1	26695	
LR133	26695 *strep*^{R}	Lab. collection
LR293, LR294	26695 *recA::Cm*	Lab. collection
LR827, LR828	26695 *dprA::Cm*	Lab. collection
LR768, LR769	26695 *comB2::Cm*	Lab. collection
LR776, LR777	26695 *comEC::Km*	Lab. collection
LR887	26695 *pUreA-GFPmut2-Km*	Lab. collection
LR901, LR902	26695 *pUreA-GFPmut2-Km comEC::Cm*	Lab. collection
LR982	26695 *pUreA-GFPmut2-Km hp1473::Cm*	This work
LR762	26695 *hp1473::Cm*	This work
LR965	26695 *hp1473::Cm rdxA ::hp1473-Km*	This work
LR1038	26695 *hp1473::Cm rdxA ::hp1473-FLAG-Km*	This work
LR1000	26695 *hp1473::Cm rdxA ::hp1473-T165A-Km*	This work
LR1051	26695 *hp1473::Cm rdxA ::hp1473-C15SC18S-FLAG-Km*	This work
LR1209	26695 *pUreA-hp1473 flag-Cm hp1473::Apra*	This work
LR1211	26695 *pUreA-hp1473 flag-Cm hp1473::Apra*	This work
LR1213	26695 *pUreA-hp1473 flag-Cm hp1473::Apra*	This work

Table S3.
Oligonucleotides used in this study

Name	Sequence (5'-3')	Description
1473 F	ATGCGCTGTTTAACCTGTTTG	hp1473 forward
1473 R	TCATTCATCCGCGCTGCAAAG	hp1473 reverse
1473 inverse R	CGGGGTACC GATTTTCACAAACTCTGCACC	--
1473 inverse F	CGCGGATCC GGCGTTTAAGGGCTAATAATGC	--
Op3	GTAATTTTTCTATGCCTTGGTTTTCTTATTCCTCCTAGTTAGTCA GGTACC	HP1473 954
Op4	CTTTGCAGCGCGGATGAATGAATGGCTAAAATGAGAATATCAC C	HP1473 KanR
Op13	GCCCTAAAAGAAGCCCTAAAATACCTTAAAAC	TA fw
Op14	GTTTTAAGGTATTTTAGGGCTTCTTTTAGGGCGGTGCCGGTGG TG	TA rev
Op302	GCTTTCT TTT AAG CCT CTT TCC CCA AAT TCC TTGAACGATTTGCCCTTAAGCTTAAAGG	C15SC18S F
Op303	CCTTTAAGCTTAAGGGCAAATCGTTCAAGGAATTTGGGGAAAG AGGCTTAAAAGAAAGC	C15SC18S R
Op247	GAGGGGTTTGTACTAGGGTTTATACGACTACCCCTAGAAAGCC TAACTCGGCTTTAAGAAAGGTTGCCAAAGTTC	Streptomycin resistant (75-mer) ssDNA for electroporation
Op611	CTTTAAGAATAGGAGAATAAGGAATTC**ATG**CGCTGTTTAACCT GTTTGAAGC	For PureA-ATG/ComFC
Op612	TTACTTATCGTCGTCATCCTTGTAATCTTCATCCGCGCTGCAAA GCGCG	Rev ComFC-FLAG*taa
Op613	GCTTCAAACAGGTTAAACAGCGCATGAATTCCTTATTCTCCTAT TCTTAAAG	Rev PureA-GAATTCATG/ComFC
Op614	GATTACAAGGATGACGACGATAAGTAAAGCGGCCGCGACTCT AGATCATAATCAGCC	For FLAG* - linker
Op853	GAGAATATTGTAGGAGATCTTCTAGAAA**GAT**AAAGAGGGCTT AAAACAGCGCTTAAGCC	For Eco47- HP1473
Op854	CCGGATGGCTCGAGTTTTTCAGCAAGATTCAGGGCGGTTACCC CCTAAACC	Rev HP1473 dw-Eco47
Op855	GGCTTAAGCGCTGTTTTAAGCCCTCTTTATCTTTCTAGAAGATC TCCTACAATATTCTC	Rev Eco47- HP1473up
Op856	GGTTTAGGGGGTAACCGCCCTGAATCTTGCTGAAAAACTCGAG CCATCCGG	For HP1473dw -Eco47
Op857	TCGCCGCTTTTATAAAATGCGCTGTTTAACCTGTTTGGTACCCG GGTGACTAACT	For HP1473up /20nt HP1473-Apra
Op858	TTAAAAAATAAAATTATAACTCATTCATCCGCGCTGCAAAGGAT CCCCGTGTCATTATT	Rev Apra- 20nt HP1473+HP1473dw

Op859	AATAATGACACGGGGATCCTTTGCAGCGCGGATGAATGAGTT ATAATTTTATTTTTTAA	For Apra- 20nt HP1473+Hp1473dw
Op860	AGTTAGTCACCCGGGTACCAAACAGGTTAAACAGCGCATTTTA TAAAAGCGGCGA	Rev HP1473up /20nt Hp1473-Apra
XV2	TGGGTGAACCTGCAGGTGGGCAAAGATGTCCTAGCAATGTAA TCGTCAAGCTTTATGCCGTT	62 mer ssDNA5-Cy5 labelled
cXV2	AACGGCATAAAGCTTGACGATTACATTGCTAGGACATCTTTGC CCACCTGCAGGTTCACCCA	62 mer ssDNA Complementary to XV2

Table S4.
Plasmids used in this study

Name	Description	Source
p978	pJet1.2-*hp1473::Cm*	This work
P1175	pJet1.2-*RdxA:: Km*	Lab collection
p1176	pJet1.2-*RdxA:: Prom-hp1473-Km*	This work
p1204	pJet1.2-*RdxA:: Prom-hp1473-T165A-Km*	This work
P1284	pJet1.2-*RdxA:: Prom-hp1473-FLAG-Km*	This work
P1310	pJet1.2-*RdxA:: Prom-hp1473-C15SC18S-FLAG-Km*	This work
P1410	pET21-*His6-TEV- hp1473*	This work
P1412	pET21-*His6-TEV- hp1473-T165A*	This work
P1088	pJet1.2-PromUreA-Cm	Lab collection
P1672	pJet1.2-PromUreA-*hp1473*-Flag-Cm	This work
P1674	pJet1.2-PromUreA-*hp1473-T165A*-Flag-Cm	This work
P1676	pJet1.2-PromUreA-*hp1473-C15S C18SFlag*-Cm	This work
P1699	pJET1.2-*hp1473::Apra*	This work
