# Supplementary material for: ComFC mediates transport and handling of single-stranded DNA during natural transformation
Source: Nat Commun. 2022 Apr 12;13:1961. doi: 10.1038/s41467-022-29494-z (PMC9005727; doi:10.1038/s41467-022-29494-z)
Supplement: Supplementary file 2 — Reporting Summary [file 41467_2022_29494_MOESM2_ESM.pdf]

nature research

Corresponding author(s): Quevillon-Cheruel and Radicella

Last updated by author(s): Mar 9, 2022

Reporting Summary

Nature Research wishes to improve the reproducibility of the work that we publish. This form provides structure for consistency and transparency in reporting. For further information on Nature Research policies, see Authors & Referees and the Editorial Policy Checklist .

Statistics

For all statistical analyses, confirm that the following items are present in the figure legend, table legend, main text, or Methods section.

n/a	Confirmed	
☐	☒	The exact sample size (*n*) for each experimental group/condition, given as a discrete number and unit of measurement
☐	☒	A statement on whether measurements were taken from distinct samples or whether the same sample was measured repeatedly
☐	☒	The statistical test(s) used AND whether they are one- or two-sided<br>*Only common tests should be described solely by name; describe more complex techniques in the Methods section.*
☒	☐	A description of all covariates tested
☒	☐	A description of any assumptions or corrections, such as tests of normality and adjustment for multiple comparisons
☐	☒	A full description of the statistical parameters including central tendency (e.g. means) or other basic estimates (e.g. regression coefficient) AND variation (e.g. standard deviation) or associated estimates of uncertainty (e.g. confidence intervals)
☒	☐	For null hypothesis testing, the test statistic (e.g. *F*, *t*, *r*) with confidence intervals, effect sizes, degrees of freedom and *P* value noted<br>*Give P values as exact values whenever suitable.*
☒	☐	For Bayesian analysis, information on the choice of priors and Markov chain Monte Carlo settings
☒	☐	For hierarchical and complex designs, identification of the appropriate level for tests and full reporting of outcomes
☒	☐	Estimates of effect sizes (e.g. Cohen's *d*, Pearson's *r*), indicating how they were calculated

Our web collection on statistics for biologists contains articles on many of the points above.

Software and code

Policy information about availability of computer code

Data collection	All data collection described in Methods. All immunofluorescence images and movies collected via NIS Elements AR 5.2 (Nikon).
Data analysis	Microscopy images were processand analyzed using NIS-element software (Nikon Corp., Tokyo, Japan), ImageJ or Volocity software. For statistical analysis GraphPad Prism software was used. Software used for crystal structure determination and analysis are described in the text.

For manuscripts utilizing custom algorithms or software that are central to the research but not yet described in published literature, software must be made available to editors/reviewers. We strongly encourage code deposition in a community repository (e.g. GitHub). See the Nature Research guidelines for submitting code & software for further information.

Data

Policy information about availability of data

All manuscripts must include a data availability statement. This statement should provide the following information, where applicable:

- Accession codes, unique identifiers, or web links for publicly available datasets
- A list of figures that have associated raw data
- A description of any restrictions on data availability

The atomic coordinates and structure factors of B2-HpComF have been deposited at the Brookhaven Protein Data Bank under the accession number 7P0H. When not present in the main text or the supplementary materials, data that support the findings are available from the correponding authors upon reasonable request.

Field-specific reporting

Please select the one below that is the best fit for your research. If you are not sure, read the appropriate sections before making your selection.

- [x] Life sciences
- [ ] Behavioural & social sciences
- [ ] Ecological, evolutionary & environmental sciences

For a reference copy of the document with all sections, see nature.com/documents/nr-reporting-summary-flat.pdf

Life sciences study design

All studies must disclose on these points even when the disclosure is negative.

Sample size	Sample sizes were deStaeirsmisisnezddauccteoxrdteinigcito commonly accepted standards in the field. The number of independent experiments and the number of cells scored (n) are indicated in the figures and figure legends.
Data exclusions	n/a
Replication	As mentioned above experiments were repeated and were only discarded when the controls failed.
Randomization	n/a
Blinding	n/a

Reporting for specific materials, systems and methods

We require information from authors about some types of materials, experimental systems and methods used in many studies. Here, indicate whether each material, system or method listed is relevant to your study. If you are not sure if a list item applies to your research, read the appropriate section before selecting a response.

Materials & experimental systems

n/a	Involved in the study	
[ ]	[x]	Antibodies
[x]	[ ]	Eukaryotic cell lines
[x]	[ ]	Palaeontology
[x]	[ ]	Animals and other organisms
[x]	[ ]	Human research participants
[x]	[ ]	Clinical data

Methods

n/a	Involved in the study	
[x]	[ ]	ChIP-seq
[x]	[ ]	Flow cytometry
[x]	[ ]	MRI-based neuroimaging

Antibodies

Antibodies used	Mouse anti FLAG Monoclonal Sigma F1804-1MG 088K6018; Rabbit anti HpComF produced in our laboratory; Rabbit anti-MotB was from Ivo Boneca's laboratory (Institut Pasteur)
Validation	The anti-ComF antibody was validated using extracts from bacterial strains mutated in the comF gene
