# Supplementary material for: ComFC mediates transport and handling of single-stranded DNA during natural transformation
Source: Nat Commun. 2022 Apr 12;13:1961. doi: 10.1038/s41467-022-29494-z (PMC9005727; doi:10.1038/s41467-022-29494-z)
Supplement: Supplementary file 4 — Description of Additional Supplementary Files [file 41467_2022_29494_MOESM4_ESM.pdf]

Title: Supplementary Movie 1
Description: Movie showing the internalisation of fluorescent tDNA into a *wild-type* bacteria. The movie is representative of 26 bacteria analysed.

Title: Supplementary Movie 2
Description: Movie showing the internalisation of fluorescent tDNA into a *ΔcomEC* bacteria. The movie is representative of 25 bacteria analysed.

Title: Supplementary Movie 3
Description: Movie showing the internalisation of fluorescent tDNA into a *ΔcomFC* bacteria. The movie is representative of 148 bacteria analysed.
